# Optimizing culture conditions for establishment of hairy root culture of *Semecarpus anacardium* L.

**DOI:** 10.1007/s13205-017-0608-x

**Published:** 2017-04-11

**Authors:** Bhuban Mohan Panda, Urmil J. Mehta, Sulekha Hazra

**Affiliations:** 1 Plant Tissue Culture Division, CSIR-National Chemical Laboratory, Pune, India; 2 Ajeet Seed Ltd, Chitegaon, Paithan, Aurangabad, Maharashtra 431105 India

**Keywords:** Hairy root culture, In vitro culture, *Semecarpus anacardium*, *rol* genes, Transformation

## Abstract

*Semecarpus anacardium* L. is a tree species which produces secondary metabolites of medicinal importance. Roots of the plant have been traditionally used in folk medicines. Different strains of *Agrobacterium rhizogenes* (A4, ATCC15834 and LBA 9402) were used for induction of hairy roots in in vitro grown tissues of the plant. Hairy root initiation was observed after 25–30 days of infection. Optimum transformation frequency of 61% was achieved on leaf explants with ATCC15834 strain. Infection time of 30 min resulted in greater transformation frequency compared to 10 and 20 min, respectively. The hairy roots cultured in growth regulator-free semi-solid woody plant medium differentiated into callus. Whole shoots infected with ATCC 15834 were found to produce more transformants upon co-cultivation for 4 (65%) and 5 (67%) days. Induction of hairy roots in stem explants infected with ATCC 15834 was lower (52%) compared to leaves (62%) after 4 days of co-cultivation. In A4 and LBA9402 strains transformation efficiency was 49 ± 2.8% and 36 ± 5.7% in shoots after 4 days of co-cultivation. Transformation frequency was higher in ATCC15834 strain, irrespective of explants. The hairy roots of *S. anacardium* elongated slowly upon transfer to half-strength liquid medium. After 3–4 passages in liquid medium slender hairy roots started differentiating which were separated from the original explants. Visible growth of the roots was observed in hormone-free liquid medium after 2–3 months of culturing. Polymerase chain reaction with gene-specific primers from *rol A*, *B* and *C* genes confirms the positive transformation events.

## Introduction

Plants have been tapped as “chemical factories” for their ability to produce phytochemicals of industrial and pharmaceutical importance from simple building blocks. The major drawback of extracting phytochemicals from normal plant tissue is that their growth and yield are significantly impacted due to environmental factors. The complex extraction procedure itself is detrimental to plant survival (Ono and Tian 2011). *Semecarpus anacardium* (Anacardiaceae), commonly known as marking nut tree, is used as an herbal drug in Ayurvedic and Unani medicines for being caustic, astringent, antirheumatic, vesicant and for being used to treat anorexia, cough, asthma, indigestion, ulcer, piles and various neurological diseases (Nadkarni 1976). These medicinal properties of *S. anacardium* L. are attributable to production of secondary metabolites (Raut et al. 2007). The roots of this plant have been used in folk medicine as anti-fertility agent for women. There is no report on chemical characteristics of the compounds present in roots.

Various aspects and applications of hairy root cultures (HRCs), include phytochemicals, recombinant protein production, phytoremediation, molecular breeding and crop improvement, introduction of desirable foreign genes, rhizosphere physiology and biochemistry, metabolic engineering, bioreactor design and general overviews of the system (Ono and Tian 2011). Nowadays, HRCs receive more attention as biological matrices for producing valuable metabolites as they have several attractive features, including high genetic stability and relatively fast growth rates. Harvesting the roots for medicinal and chemical uses is destructive for the plants and hence there has been increasing interest in developing hairy root cultures from several medicinal plant species (Zhou et al. 2011).

Initiation and establishment of HRCs in *S. anacardium* could lead to development of an independent in vitro system. This can be used to study the production and identification of chemical compounds in controlled environments. The present investigation was conducted to optimize conditions for establishment of hairy root culture of *S. anacardium*. These experiments reports, establishment of hairy root culture using *A. rhizogenes* ATCC15834 from leaf explants. Virulence of different strains (ATCC 15834, A4 and LBA9402) and susceptibility of different explants (leaf, stem (shoot without leaves) and shoots) were optimized. Compatibility of bacterial strain and explant type was discussed. Finally, confirmations of transformation by PCR using *rol* specific primers were described.

## Materials and methods

### Bacterial strains

Three wild-type agropine strains of *A. rhizogenes* (ATCC15834, A4 and LBA9402) were used for transformation. *A. rhizogenes* ATCC15834 were kindly provided by Prof. Sujata Bhargava (Department of Botany, University of Pune) for preliminary investigation of transformation procedure. Strains A4 and LBA9402 were obtained from Prof. Sumita Jha (Center of Advanced Studies in Cell and Chromosome Research, Department of Botany, University of Calcutta, Kolkata).

### Bacterial media and growth

Yeast mannitol broth (YMB) medium was used for bacterial culture. Fresh bacterial culture was initiated from glycerol stock. The bacteria were streaked on nutrient YMB agar (15 g/l) medium and incubated for 48 h at 27 °C. Single colony of the *A. rhizogenes* was picked up and inoculated in 10 ml of YMB nutrient broth. The culture was incubated at 27 °C on rotary shaker at 120–130 rpm. Bacterial culture with approximate optical density (1.0) at 600 nm was used for infecting different explants of *S. anacardium*.

### Plant material

Tissues from seedling-derived in vitro shoot cultures of *S. anacardium* were used as explants (Panda and Hazra 2010). Leaves, stems and shoots were isolated from axenic cultures maintained in charcoal incorporated WPM medium (Lloyd and McCown 1980).

### Infection and co-cultivation of explants

Isolated leaves, stems (shoots without leaves) and whole shoots were infected with different strains of *A. rhizogenes* ATCC15834, A4 and LBA9402. These explants were transferred aseptically to 90 mm Petri dishes containing 20 ml of bacterial suspension. For experimental control the bacterial suspension was substituted with YMB medium. All the explants were pricked with the help of sterile hypodermic syringe needle, Dispovan (Hindustan Syringes and Medical Devices Ltd, Haryana, India). In the first experiment *A. rhizogenes* ATCC15834 was used to optimize duration of infection and co-cultivation period with leaf explants. In the second experiment, *A. rhizogenes* A4 and LBA9402 strains were tested along with *A. rhizogenes* ATCC15834 in conjunction with different explants including leaves, stems and shoots. The wounded explants were removed from bacterial suspension, blotted dry on filter paper and cultured on half-strength semisolid WPM basal medium in Petri dishes. The cultures were co-cultivated in light for different periods of time duration ranging from 1 to 7 days. Explants from various time periods of co-cultivation were washed in antibiotic solution of cefotaxime (Alkem, India) (400 mg/l) to eliminate remaining *A. rhizogenes*. The explants were transferred to half-strength WPM basal medium containing 400 mg/l cefotaxime after drying on filter paper. Explants from the control cultures were also treated similarly. Ten to fifteen explants were inoculated in each plate with 3–4 plates per replicate. All experiments were done in triplicate. Observations were recorded after 8 weeks of culture. Concentration of cefotaxime in semi-solid media was halved each week and finally, cultures free of *A. rhizogenes* were transferred to half-strength WPM without antibiotics. Explants with single root or root cluster were noted for transformation. The frequency of infection was determined. DNA isolated from untransformed roots that were induced from shoot cultures served as negative control.

All cultures were incubated at 25 ± 2 °C temperature in 16 h photoperiod under diffuse cool white fluorescent lights (50 µmol/m^2^/s). The observed data were subjected to Analysis of Variance (ANOVA). Graphs were plotted using Origin 6.1 software.

### DNA isolation

Genomic deoxyribose nucleic acid (DNA) was extracted from the putatively transformed and untransformed roots (from shoot cultures) following the protocol of Khanuja et al. (1999).

### Plasmid DNA isolation

Plasmid DNA from the *A. rhizogenes* strains was isolated using standard alkaline lysis method (Sambrook et al. 1998). Quantitative and qualitative estimation was also done with spectrophotometer. For visual estimation of quantity, plasmid DNA was loaded in 0.8% agarose gel with λ DNA as standard.

### Primers used for screening of *rol* genes

Transformants were screened for the presence of *rol A, B* and *C* genes using the sequence specific primers. The primers were custom synthesized from MWG-Biotech, Bangalore, India.

- For *rol A* gene: For-5′-CAGAATGGAATTAGCCGGACTAA-3′
- Rev-5′-CGTATTAATCCCGTAGGTTTGTTT-3′
- For *rol B* gene: For-5′-ATGGATCCCAAATTGCTATTCCCCCACGA-3′
- Rev-5′-TTAGGCTTCTTTCATTCGGTTTACTGCAGC-3′
- For *rol C* gene: For-5′-CATTAGCCGATTGCAAACTTG-3′
- Rev-5′-ATGGCTGAAGACGACCTG-3′

### PCR condition

The PCR reactions were carried out in 25 μl volume and consist of 40 ng of DNA, 10 pm/μl primer, 200 mM dNTP, 1 U of Taq DNA polymerase, 1× PCR buffer and 1.5 mM MgCl_2_. All the PCR components were from Genei, Bangalore. Amplification of DNA was performed in a thermal cycler (Veriti thermal cycler, Applied Biosystems) using the following sequence: initial denaturation at 94 °C for 5 min, followed by 35 cycles of 94 °C for 1 min, annealing 52.5 °C (*rolB* gene)/62 °C (*rolA* and *C* gene) for 1.5 min and 72 °C for 2 min followed by final extension at 72 °C for 10 min. The amplification products were visualized on 1.5% w/v agarose gel stained with ethidium bromide.

## Results and discussion

Factors that influence successful transformation in plant tissue and hairy root induction include genotype, species, age, type of plant tissue (Sevon et al. 2002), type of *Agrobacterium* strain, density of the bacterial suspension (Park and Facchini 2000), time duration of infection and media composition (Nourozi et al. 2016). A number of chemicals may also promote these processes, e.g., acetosyringone (Joubert et al. 2002).

### Infection of leaf explants with *A. rhizogenes* ATCC15834

Optimum transformation frequency of 61% was noted in explants of *S. anacardium* L. infected for 30 min in the bacterial suspension (Table 1). Explants with 10 min of infection did not show any transformation except on the 4th day of co-cultivation. Varying percentages of transformation were observed within 20 min of infection (Table 1). Researchers have reported effect of infection time on transformation frequency using *A. rhizogenes* which depends on plant species. Five minutes of infection to wounded explants was effective in inducing hairy roots in *Datura tatula* L. (Peng et al. 2008), *Papaver bracteatum* Lindl. (Rostampour et al. 2009), *Echinacea* sp. (Romero et al. 2009) in *Linum mucronatum* (Samadi et al. 2012) and in *Agastache foeniculum* (Nourozi et al. 2016), whereas in *Silybum marianum* (Rahnama et al. 2008) and in *Fagopyrum tataricum* (Thwe et al. 2016), 10 min of infection was optimum. The infection time was 20 min in *Artemisia annua* (Giri et al. 2001) and in *Portulaca oleracea* (Moghadam et al. 2014). In glycine *max* 45 min of infection was required (Liu et al. 2008) to get successful transformation.

**Table 1:** Effect of infection time on transformation frequency (%) of *A. rhizogenes* ATCC15834 with leaves as explants

Days of co-cultivation	Transformation frequency (%) (mean ± SD)		
	Time duration of infection		
	10 min	20 min	30 min
1	00 ± 00 (135)	00 (115)	00 ± 00 (130)
2	00 ± 00 (125)	23 ± 1.4 (106)	35 ± 2.12 (140)
3	00 ± 00 (98)	28 ± 4.12 (106)	49 ± 3.5 (128)
4	7 ± 00 (122)	39 ± 2.5 (123)	61 ± 2.8 (110)
5	00 ± 00 (140)	25 ± 2.8 (90)	53 ± 2.12 (95)
6	00 ± 00 (102)	15 ± 2.12 (145)	42 ± 4.2 (145)
7	00 ± 00 (110)	7 ± 1.4 (120)	29 ± 2.12 (116)
ANOVA	–	Sig 1%	Sig 1%

Figures in parenthesis () indicates number of replicates

**Fig. 1:**
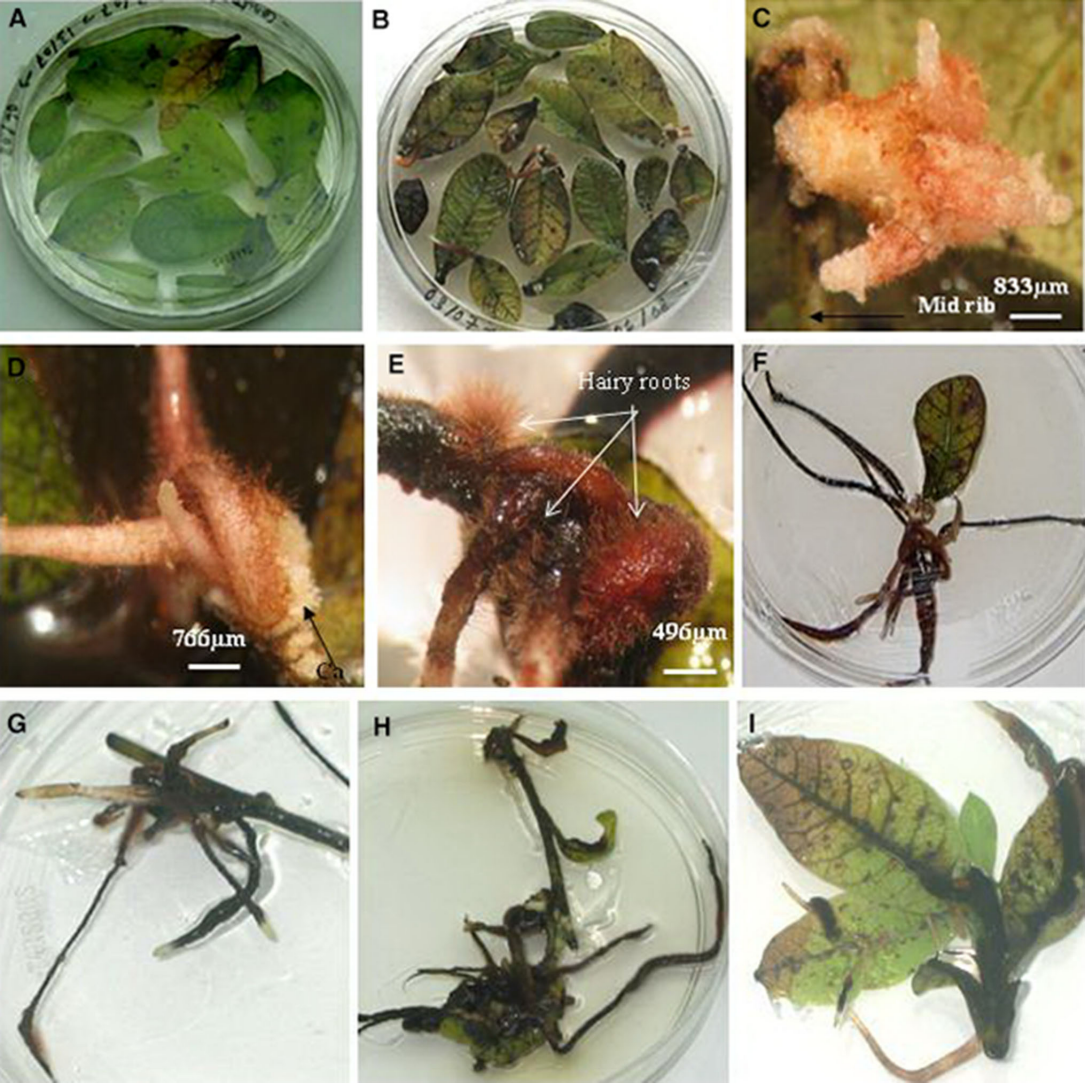
Induction of hairy roots in different explants. **a** Control leaf explants in half-strength WPM medium without any root development after 8 weeks of culturing in antibiotic-incorporated medium. **b** Induction of root in leaves infected with *A. rhizogenes* after 3–4 weeks of culturing in half-strength WPM medium containing antibiotics. **c** Initiation of hairy root-like structure originating from the mid rib region of leaf explants in half-strength WPM medium containing antibiotics. **d** Cluster of roots with fine root hairs arising from the leaf explants in half-strength WPM medium containing antibiotic; the base of the roots are associated with callus. **e** Thin slender roots with root hairs developed from the clusters of roots on culturing in GR-free half-strength WPM medium. **f** Cluster of hairy roots showing growth and elongation from leaf explants infected with *A. rhizogenes* cultured in half-strength WPM medium. **g** Initiation and growth of hairy roots from stem explants cultured in half-strength WPM medium. **h** Hairy roots in stem explants originate mostly from leaves attached to shoot explants infected with *A. rhizogenes* in half-strength WPM medium. **i** Emergence of hairy roots from the mid rib region of leaf explants attached to shoot

In *S. anacardium* varied transformation frequency was observed when the leaf explants were co-cultivated for different durations. Optimum transformation was achieved in explants kept for 4 days of co-cultivation on growth regulator-free medium (Table 1). In *Rehmannia glutinosa* (Hwang 2009) the co-cultivation period was 1 day to achieve 46.7% transformation. Two days of co-cultivation was effective to obtain 59% of transformants in *Saponaria vaccaria* L. (Schmidt et al. 2007), 60% in *L. mucronatum* (Samadi et al. 2012), and 75% in *Arachis hypogaea* L. (Kim et al. 2008). *S. marianum* (Rahnama et al. 2008) *Tylophora indica* (Chaudhuri et al. 2005) and *Gmelina arborea* Roxb (Dhakulkar et al. 2005) needed 3 days of co-cultivation to get optimum transformation frequency of 30, 60 and 32%, respectively, using *Agrobacterium rhizogenes* strains. Optimum transformation frequency of 45% was achieved in *Chonemorpha fragrans* (Moon) Alston, after 7 days of co cultivation (Kedari and Malpathak 2014). In the present study there was no root formation in control leaf explants treated with YMB medium (Fig. 1a). However, in *Semecarpus* the leaf explants co-cultivated with *A*. *rhizogenes* for 3–4 days showed successful root induction after 25–30 days of culture in antibiotic-supplemented medium over three passages of 7 days each (Fig. 1b). Root induction was observed frequently in *Agrobacterium-*infected leaves of *Semecarpus* injured along the midrib. Differentiation of roots from the wounded sites in midrib region of leaf was associated with callusing (Fig. 1c). Callusing from the wounded sites of seedling explants of California poppy (Park and Facchini 2000) and nodes/internodal explants in *T. indica* (Chaudhuri et al. 2005) in response to *Agrobacterium* had been reported. Hairy root development was confined to wounded site of explants in *Rubina tinctorum* (Ercan et al. 1999), *Papaver somniferum* (Park and Facchini 2000) and in *A. hypogaea* (Kim et al. 2008). Roots with hairy structures elongated and callusing at the base of the roots were observed in some of the cultures (Fig. 1d). It has been reported that cell division in the host target explants tissue is a prerequisite for successful *Agrobacterium* transformation (Binns and Thomashow 1988). Thin slender roots with root hairs developed from the clusters of roots on culturing in GR-free half-strength WPM (Fig. 1e). Transformed roots grew in cluster from the midrib region of the leaves on culturing in half-strength basal medium without antibiotics for 12 weeks (Fig. 1f).

### Virulence of different *Agrobacterium* strains (A4, ATCC15834 and LBA9402) and susceptibility of explants (leaf, stem and shoot)

Experiments were extended to determine the optimum co-cultivation period and efficacy of different bacterial strains to induce hairy roots in different explants. Varying root induction with different period of co-cultivation was observed in leaf explants infected with *A. rhizogenes* strains. In leaf explants of *S. anacardium* optimum rooting frequency was 62 ± 2.12%, infected with strain ATCC15834 and co-cultivated for 4 days was observed. The results concur with earlier observation of transformation in leaves. Strains A4 and LBA9402 induce rooting in 59 ± 4.94 and 42 ± 12.02% leaf explants, respectively, during the same co-cultivation period (Table 2).

**Table 2:** Effects of co-cultivation duration and bacterial strains on transformation frequency (%) of different explants

Bacterial strains	Transformation frequency (%) (mean ± SD)						
	Days of co-cultivation						
	1	2	3	4	5	6	7
Leaf explants							
Control	00 ± 00 (96)	00 ± 00 (112)	00 ± 00 (110)	00 ± 00 (133)	00 ± 00 (123)	00 ± 00 (98)	00 ± 00 (129)
A4	00 ± 00 (98)	32.5 ± 11 (120)	47 ± 8.9 (123)	58.5 ± 5 (128)	46.5 ± 9 (112)	30 ± 4.24 (120)	27 ± 4.3 (142)
ATCC15834	00 ± 00 (96)	30 ± 4 (116)	51.5 ± 2.2 (148)	61.5 ± 2 (132)	53 ± 2 (134)	40 ± 2.82 (143)	28 ± 2.8 (114)
LBA9402	00 ± 00 (98)	31 ± 11 (125)	41.5 ± 11 (120)	41.5 ± 12 (147)	35.5 ± 4 (125)	31 ± 9.19 (150)	23 ± 9.89 (114)
ANOVA		NS	Sig 5%	Sig 1%	Sig 1%	Sig 1%	Sig 1%
Stem explants							
Control	00 ± 00 (96)	00 ± 00 (106)	00 ± 00 (112)	00 ± 00 (132)	00 ± 00 (95)	00 ± 00 (96)	00 ± 00 (124)
A4	00 ± 00 (90)	31 ± 18 (103)	32.5 ± 11 (113)	45 ± 7 (134)	52 ± 3 (96)	40 ± 3 (95)	39 ± 1.4 (123)
ATCC15834	00 ± 00 (93)	35 ± 3 (123)	43.5 ± 4 (120)	52 ± 3 (122)	60 ± 9 (103)	44 ± 3 (116)	37 ± 5.6 (143)
LBA9402	00 ± 00 (92)	32 ± 10 (121)	29.5 ± 12 (94)	39 ± 2 (110)	42 ± 1 (107)	35 ± 4 (112)	29 ± 8.4 (134)
ANOVA		NS	Sig 5%	Sig 1%	Sig 1%	Sig 1%	Sig 1%
Shoot explants							
Control	00 ± 00 (112)	00 ± 00 (116)	00 ± 00 (128)	00 ± 00 (134)	00 ± 00 (102)	00 ± 00 (122)	00 ± 00 (136)
A4	00 ± 00 (122)	20 ± 10 (110)	37 ± 28.8 (135)	49 ± 3 (121)	39 ± 11 (112)	41 ± 17 (127)	44 ± 3.6 (98)
ATCC15834	00 ± 00 (124)	32 ± 3 (123)	43 ± 15.3 (138)	65 ± 10 (118)	67 ± 3 (134)	63 ± 12 (146)	58 ± 8 (95)
LBA9402	00 ± 00 (113)	17 ± 8 (116)	27 ± 15.3 (121)	36 ± 5.7 (118)	33 ± 7.6 (126)	32 ± 8 (133)	27 ± 12 (104)
ANOVA	–	Sig 1%	Sig 1%	Sig 1%	Sig 1%	Sig 1%	Sig 5%

Figure in parenthesis () indicates number of replicates

Induction of hairy roots in stem explants was (52%) compared to leaves (62%) after 4 days of co-cultivation. Rooting frequency increased to 60 ± 9% in stem explants after 5 days of co-cultivation. Like leaf explants, stem explants also exhibited higher rooting as well as the transformation frequency when infected with strain ATCC15834. Transformed roots originated in cluster from stem explants cultured in half-strength WPM without GR (Fig. 1g). These roots grew slowly and covered the surface of solid media in petriplates. Optimum hairy root induction of 67 ± 2.3% in shoot explants was noted in shoot explants cocultivated for 5 days with *A*.*Rhizogenes* ATCC 15834. whereas 65% of root initiation was observed in 4 days cocultivations. Transformation frequency with A4 and LBA9402 strains was 49 ± 2.8 and 36 ± 5.7%, respectively, in shoots co-cultivated for 4 days. *A. rhizogenes* strain ATCC15834 was found to be more virulent in all the three types of explants. Strain ATCC15834 was more potent in infecting cotyledon explants of *Rubia tinctorum* (Ercan et al. 1999) in comparison to strains 2628, R1000 and 9365. This strain was one of the potent *A. rhizogenes* for transformation in *Capsicum frutescens* (Setamam et al. 2014). Roots in clusters (Fig. 1h) arose from the wounded sites of intact leaves of shoots. The root induction location was mainly concentrated in the midrib region of leaves (Fig. 1i). Rooting from the veins of leaf in *Pueraria phaseoloides* infected with *A. rhizogenes* ATCC15834 was noted (Shi and Kintzios 2003).

To summarize the data generated in this experiments, *A. rhizogenes* ATCC15834 is the best strain and shoots are the most suitable explants. The optimum transformation frequency in shoot explants of *S. anacardium* may be ascribed to the presence of intact leaves in addition to stem. Shoots are the explants of choice for hairy root induction using *A*. *rhizogenes* in apple rootstock Jork 9 (Pawlicki-Jullian et al. 2002), *P. bracteatum* Lindl. (Rostampour et al. 2009) and in *T. indica* (Chaudhuri et al. 2005). Virulence or infectivity of *Agrobacterium* strains varies among plant hosts and explant types (Hobbs et al. 1989; Bush and Pueppke 1991; Baranski et al. 2006). High frequency of hairy root induction in leaf explants of *Pelargonium* sp. and *Psoralea corylifolia* compared to internodes and petioles was observed by Saxena et al. 2007 and Rajkumar and Murugesan, 2014, respectively.

Transformation efficiency in plant species can diverge between different bacterial strains (Godwin et al. 1991; Hu and Alfermann 1993). Optimum hairy root initiation was reported in shoot tips of *A. annua* infected with *A*. *rhizogenes* LBA9402 than strain A4, ATCC15834, 9365 and 9340 (Giri et al. 2001). Similarly, in leaf explants of *Saponaria vaccaria* L., strain LBA9402 was found to be more potent than *A*. *rhizogenes* ATCC15834 for infection (Schmidt et al. 2007). *A*. *rhizogenes* A4 is reportedly most potent for dicotyledonous plants (Kuzovkina and Schneider 2006). In leaf explants of *Pelargonium* sp., 100% transformation frequency was reported with strain A4 (Saxena et al. 2007).

**Fig. 2:**
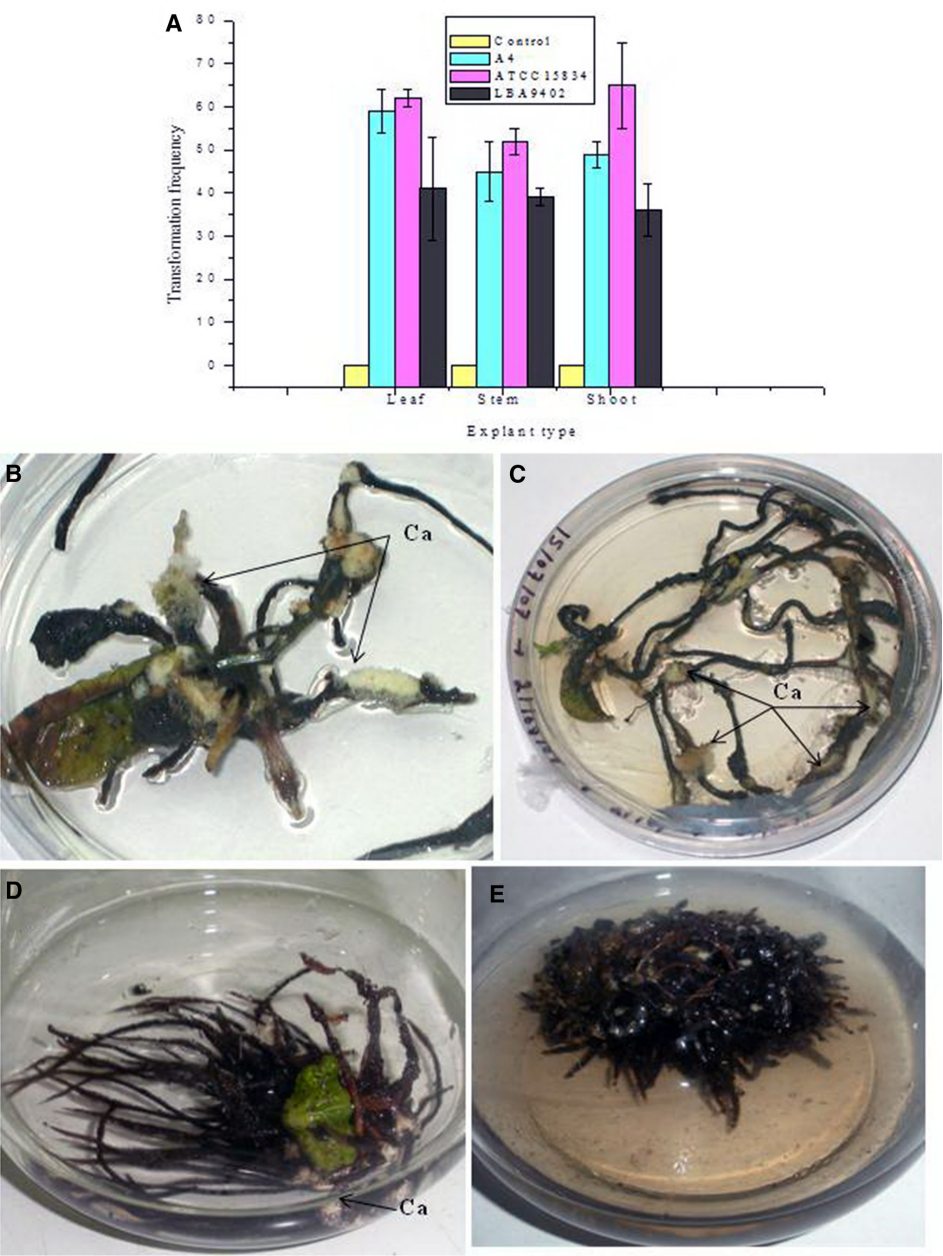
Comparison of hairy root induction in different explants, callusing of hairy roots in solid medium and hairy root culture in liquid medium. **a** Comparison of different *A*. *rhizogenes* strain and explant type after 4 days of co-cultivation periods on transformation frequency of *S. anacardium*. **b**, **c** Hairy roots dedifferentiated into callus during culturing after limited growth in semi-solid half-strength WPM medium. **d** Hairy root culture in liquid medium, the original leaf explant was still attached, with dedifferentiation of the roots into callus. **e** Clump of hairy roots cultured in liquid medium, the medium become brown due to leaching of phenolics compound

In *S. anacardium* HRCs, root thickening and dedifferentiation into callus were observed on further culturing in semi-solid medium (Fig. 2b, c). Shorter and thicker root development has been observed in apple rootstock jork9 with *A*. *rhizogenes* ATCC15834 (Pawlicki-Jullian et al. 2002). The hairy roots of *S. anacardium* elongates on transferring to half-strength liquid medium. But the elongation rate was very slow in thicker roots (data not shown). After 3–4 passages in liquid medium thin hairy roots started differentiating slowly which were separated from the original explants. Visible growth of the roots was observed in liquid medium after 2–3 months of culturing (Fig. 2d, e). The slow growth of HRCs in *S. anacardium* could be due to the presence of excess growth inhibitory phenolics in the culture or due to slow growth habit of tree sp. in vitro.

The browning of the culture medium was indicative of leaching of phenolics (Fig. 2c, e). Further biochemical studies are required to reveal the nature of phenolics compounds. Callus formation from the roots in liquid medium was noted, and similar differentiation of the roots was already observed in semisolid medium. Callusing in hairy roots cultured in GR-free medium was reported in *D. tatula* L. (Peng et al. 2008) and was assigned due to high capacity of the tissue to dedifferentiate resulting in callus formation. Callus formation was also observed in the hairy roots of *Bacopa monnieri* induced by *A. rhizogenes* LBA9402 (Majumdar et al. 2011). Large numbers of *A. rhizogenes*-mediated hairy root lines were produced in *S. anacardium* L. but only very few of those gave rise to HRCs. This could be due to the different sites of insertion of T-DNA as well as the copy number of transgenes. In addition, deletions and/or duplications of T-DNA sequences or target site sequences may also lead to variation in the hairy root clones (Dhakulkar et al. 2005).

### Confirmation of transgenic status

**Fig. 3:**
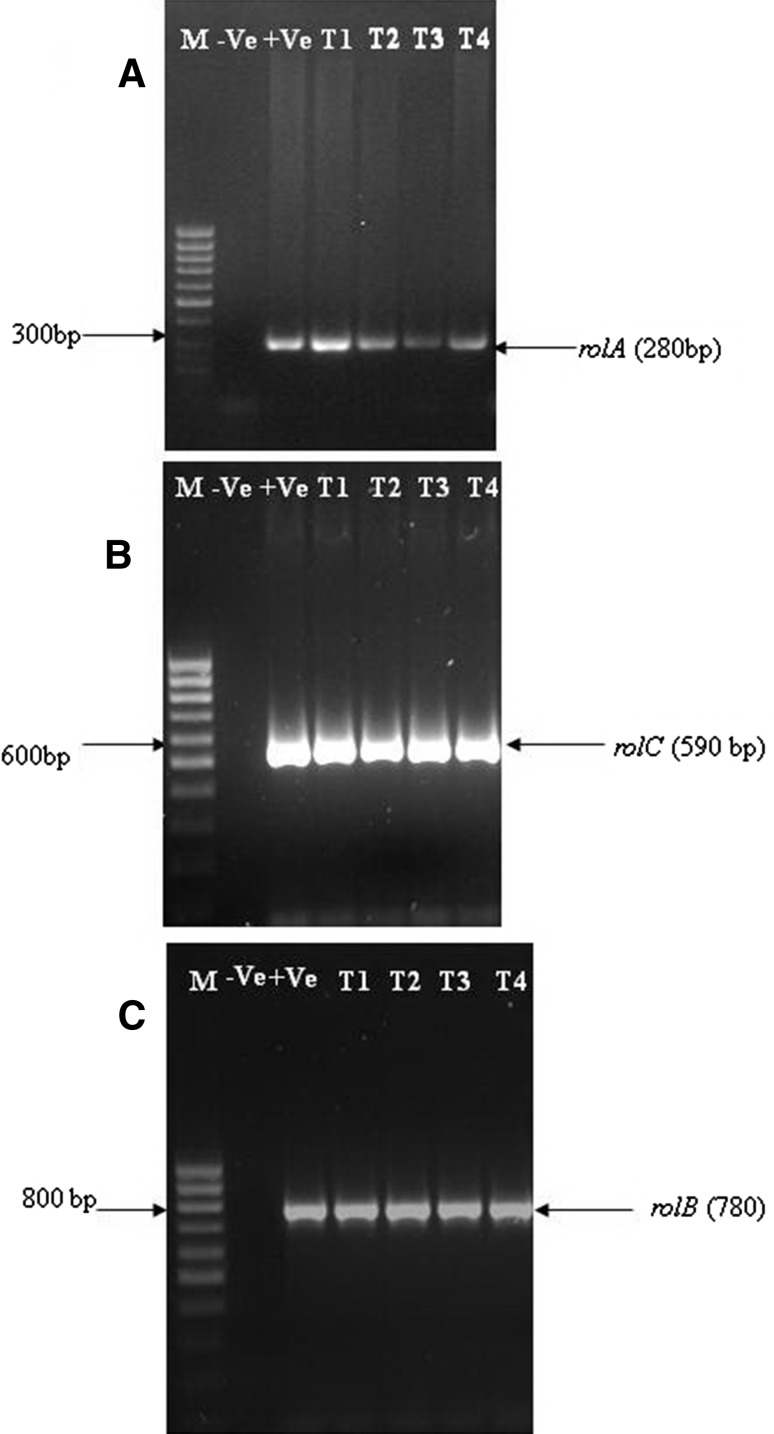
Confirmation of transformation by PCR. **a** PCR amplification of 280 bp fragment of the *rolA* gene. *Lane 1* molecular weight marker (100 bp ladder); *lane 2* DNA from non-transformed roots (negative control); *lane 3 A. rhizogenes* plasmid DNA (positive control); *lane 4* sample 1; *lane 5* sample 2; *lane 6* sample 3; *lane 7* sample 4. Samples 1, 2, 3 and 4 were DNA of transformed roots obtained after *A. rhizogenes* infection. **b** PCR amplification of 590 bp fragment of the *rolC* gene. *Lane 1* molecular weight marker (100 bp ladder); *lane 2* DNA from non-transformed roots (negative control); *lane 3 A. rhizogenes* plasmid DNA (positive control); *lane 4* sample 1; *lane 5* sample 2; *lane 6* sample 3; *lane 7* sample 4. Samples 1, 2, 3 and 4 were DNA of transformed roots obtained after *A. rhizogenes* infection. **c** PCR amplification of 780 bp fragment of the *rolB* gene. *Lane 1* molecular weight marker (100 bp ladder); *lane 2* DNA from non-transformed roots (negative control); *lane 3 A. rhizogenes* plasmid DNA (positive control); *lane 4* sample 1; *lane 5* sample 2; *lane 6* sample 3; *lane 7* sample 4. Samples 1, 2, 3 and 4 were DNA of transformed roots obtained after *A. rhizogenes* infection

However, due to the plasticity of the plant cells roots could be induced in explants in condition other than that of transformation. Therefore, efforts were made to confirm the presence of *rol* genes using PCR. Confirmation of transgenic status of the tissue was done with PCR amplification of the DNA isolated from the hairy roots using forward and reverse primers of *rol* genes (*rolA*, *rolB* and *rolC*). Plasmids from *A. rhizogenes* served as the positive control, and DNA from the non-transformed roots of shoot culture served as the negative control. All transformants (infected with different strains) showed the presence of the *rolA* (300 bp) (Fig. 3a), *rolB* (780 bp) (Fig. 3b) and *rolC* (590 bp) (Fig. 3c) in DNA-amplified product, confirming the transgenic nature of the hairy root lines.

## Conclusion

In the present investigation hairy root cultures were established from an important medicinal woody tree *S. anacardium*. Induction of hairy root occurs at frequency of 67 and 62% in shoot and leaf explants, respectively. There was a significant difference in hairy root induction with respect to the type of explants and bacterial strains used. Shoot was found to be the explant of choice for hairy root induction compared to stem and leaves. Among the bacterial strains tested ATCC15834 was found to be the most virulent than A4 and LBA9402. These are some of the preliminary findings regarding hairy root culture for *S. anacardium*. Further research needs to be done for isolation, identification and scaling up of novel secondary metabolites in hairy root cultures.

## Abbreviations

WPM: Woody plant medium
PCR: Polymerase chain reaction
HRC: Hairy root culture
GR: Growth regulators
DNA: Deoxyribose nucleic acid
YMB: Yeast mannitol broth
